# Pen-on-Paper Label-Free Surface-Enhanced Raman Scattering (SERS) Detection of Tetracycline in Milk

**DOI:** 10.1021/acsomega.5c01637

**Published:** 2025-06-06

**Authors:** Alida Russo, Martina Piletti, Aidan J. Quinn, Daniela Iacopino

**Affiliations:** Tyndall National Institute, 8795 University College Cork, Lee Maltings Complex, Dyke Parade, T12R5CP Cork, Ireland

## Abstract

Monitoring food quality requires the development of low-cost and sensitive analytical tools with label-free and point-of-site detection capabilities. Surface-enhanced Raman scattering (SERS) is a powerful analytical technique that combines fingerprint detection capabilities with high sensitivity. In this paper, we present the pen-on-paper fabrication of SERS substrates, whereby plasmonic nanoinks were written on a paper substrate and used for the detection of food contaminant residues. To construct sensitive and robust SERS sensors, four different nanoinks with different plasmonic properties were synthesized and tested on eight papers of different weights, textures, and hydrophilicities. The combination of silver nanoink and Bristol printing paper resulted in the strongest SERS substrate, which was used for crystal violet (CV) and tetracycline detection. Concentrations as low as 10^–12^ M were obtained for CV and 2.5 ppm of tetracycline in milk, showing the application potential of these paper-based SERS sensors for label-free detection of residuals in complex food matrices.

## Graphical abstract

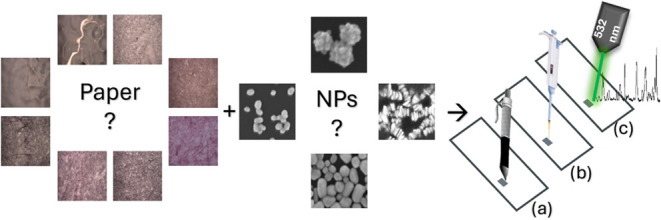

## 1 Introduction

The demand for dairy products has increased in recent years due to their nutritional value and population growth, necessitating the implementation of practices to ensure product quality and safety.^1^ Milk, especially cow’s milk, is one of the main products of the dairy industry. Poor agricultural, veterinary, and hygienic practices can lead to milk adulteration and the unacceptable presence of chemical residues. For example, antibiotic residues can be found in milk due to their widespread use in the treatment of bacterial infections in dairy cows, such as mastitis. The presence of antibiotic residues in milk is associated with many health issues, including allergic reactions (especially in hypersensitive people), disruption of Ca^2+^ metabolism (problematic for teeth and bone formation in children), photosensitivity, and increased hypersensitivity to light.^2^ Furthermore, residual antibiotics can accumulate long-term in the human body with consequent harmful effects, such as hepatotoxicity, nephrotoxicity, cytotoxicity, and potential carcinogenicity.^3^ In addition to the above health issues, the inappropriate and widespread use of antibiotics in food animals (about 73% of worldwide used antimicrobials^4^) contributes to the increase of antimicrobial resistance (AMR), which has been declared by the World Health Organization (WHO) as one of the top health challenges facing humanity in the 21st century.^5,6^ To address all these issues, as part of the Farm to Fork Strategy, the European Commission has targeted a 50% reduction in the overall sales of antibiotics for farmed animals and aquaculture by 2030 (compared to 2018).^7^ In parallel, for all antibiotics permitted for animal use, the European Commission has set maximum residue limits (MRLs) for animal-derived foods, such as milk,^8^ to ensure safe consumption.

Among antibiotics, tetracyclines are widely used to treat different bovine bacterial infections, accounting for 23.5% of the overall sales of antibiotics in Europe.^9^ Tetracyclines work through inhibition of protein synthesis in bacteria.^10^ Following a parenteral dose, tetracyclines are eliminated in milk, with traces present up to 48 h after administration. For this reason, the MRL for tetracyclines has been set to 100 μg kg^–1^ (0.1 ppm = 100 ppb).^8,11^ Considering the widespread use of tetracyclines, it is imperative to develop fast and low-cost technologies to efficiently monitor their trace presence in milk. Conventional analytical approaches, such as liquid chromatography,^12,13^ liquid chromatography-tandem mass spectrometry,^14,15^ or gas chromatography,^16^ have been widely used in the past decades to detect tetracycline residues. Despite their high reliability, these methods are tedious and require expensive instruments, complex sample preparation, and trained personnel. Other simpler methodologies have also been used, such as microbial inhibition assays,^17^ aptasensors,^18^ and immunoassays, both enzyme-linked immunosorbent assays (ELISA) and immunochromatographic strips.^19,20^ Although some of these methods allow on-site detection, it would be desirable, convenient, and lower cost to use a label-free analytical approach.

Surface-enhanced Raman scattering (SERS) is a powerful label-free analytical tool that combines the fingerprinting capabilities of Raman spectroscopy with the high sensitivity offered by the incorporation of noble metal nanostructures. In SERS, the close assembly of noble metal nanoparticles (NPs) on a substrate is engineered to create so-called hotspot areas.^21^ The Raman response of analytes deposited in close proximity to the hotspots is enhanced by a factor of up to 10^8^–10^12^ due to a combination of two mechanisms: electromagnetic (EM) and chemical (CE) enhancement.^22^ Indeed, these unique characteristics have made SERS a technique that has been widely used in different fields, from the biomedical to pharmaceutical sectors, as well as in food safety for the authentication of contaminants and microorganisms.^23^ The effectiveness of SERS depends on the type of metal nanoparticles used, their size, shape, and assembly, as well as on the substrate choice.^24^ Recent research has focused on the development of substrates featuring flexibility, low cost, and environmental friendliness, combining a good level of SERS sensitivity and selectivity with fast and low-cost fabrication.^25^ Among the various substrates, paper has been identified as a good candidate for point-of-care testing, and it has been shown to be a desirable substrate for different SERS applications, including food safety.^2,26,27^ Recently, a convenient pen direct-writing approach has been developed for the fabrication of paper-based substrates, as demonstrated by Polavarapu et al.^28^ This approach is based on the development of nanoinks (metallic nanoparticle inks) that are “written” on a paper substrate using a commercially available fountain pen. The simplicity, versatility, and intrinsic do-it-yourself (DIY) applicability of this method have the potential to expand the usability of SERS paper strips in many settings and to resource resource-limited laboratories.

In this paper, a pen-on-paper SERS approach was used to demonstrate the detection of residual tetracycline in milk matrices. A careful investigation and selection of different paper substrates and nanoinks with different spectroscopic properties were carried out to select the best paper–ink combination for this purpose. Mechanically robust Bristol paper and silver nanoparticle inks were identified as the best combination for the developed paper-based label-free SERS sensor. This study highlights the applicability of SERS for the analysis of milk contaminants, with its low cost, simplicity, and minimal sample preparation. The DIY application of nanoinks allows the fabrication and use of such diagnostic strips in third-world countries or locations with limited availability of expensive fabrication equipment and trained personnel, and opens the possibility to analyze different contaminants in complex matrices.

## 2 Experimental Section

### 2.1 Chemicals and Materials

Tetrachloroauric­(III) acid (HAuCl_4_, ≥99.9% trace metals basis), silver nitrate (AgNO_3_, ACS reagent, ≥99.0%), trisodium citrate dihydrate (ACS reagent, ≥ 99.0%), hydroquinone (Reagent Plus­(R), ≥99%), poly­(vinylpyrrolidone) (PVP, weight-average molecular weight *M*_w_ ≈ 29 000 g mol^–1^), sodium borohydride (NaBH_4_, ≥98.0%), and hydrogen peroxide (30% w/w) (H_2_O_2_, puriss. P.a., reag. ISO, reag. Ph. Eur.) were purchased from Sigma-Aldrich (Arklow, Ireland). All solutions were prepared using Milli-Q water (resistivity of 18.2 MΩ·cm). All glassware used for the synthesis of the nanoparticles was cleaned thoroughly with aqua regia and finally rinsed with Milli-Q water. The two analytes, crystal violet (CV, dye content ≥90%) and tetracycline hydrochloride (European Pharmacopoeia, high-performance liquid chromatography (HPLC) assay, ≥95%), were also bought from Sigma-Aldrich (Arklow, Ireland). Dawn Fresh Milk was purchased from a local store and produced in Ireland by Clona Dairy Products Ltd. (Clonakilty, Co. Cork).

The Parker fountain pens (Vector model, fine nib) and their standard ink cartridges were acquired at the “Cork Art Supplies” shop (Cork, Ireland). Similarly, the paper substrate Strathmore-Bristol 270 g m^–2^ (Wisconsin, USA) was purchased in the same shop.

### 2.2 Synthesis of Colloidal Solutions of Gold and Silver Nanoparticles

Following the Turkevich–Frens method,^29,30^ spherical gold nanoparticles (Au NPs) with a localized surface plasmon resonance (LSPR) at about 527 nm were synthesized by citrate reduction of tetrachloroauric­(III) acid. Specifically, 100 mL of 0.01% w/v tetrachloroauric acid solution was boiled under vigorous stirring. As soon as boiling was reached, 1 mL of 1% w/v sodium citrate was added to the solution. The boiling temperature and stirring conditions were maintained until the desired color transition from pale yellow to brilliant red was obtained (ca. 10 min). After cooling to room temperature, the colloidal solution was stored at 4 °C for further use.

A blue colloidal solution of flower-shaped gold nanoparticles (F-Au NPs) with an LSPR of ca. 618 nm was synthesized by a seeding growth approach, following a method reported by Di Nardo et al.^31^ Initially, spherical Au NP seeds (LSPR at about 518 nm) were synthesized by the citrate reduction method, adding 750 μL of sodium citrate 1% w/v to boiling and stirring 30 mL of solution 0.01% w/v of HAuCl_4_. Then, 10 mL of Milli-Q water was stirred in a 250 mL flask together with 75 μL of 1% w/v tetrachloroauric acid, 22 μL of 1% w/v sodium citrate, and 84 μL of gold seeds (with an optical density (OD) = 1). After 2 min of stirring, 1 mL of 30 mM hydroquinone was rapidly added to the solution, causing a color change to blue. The solution was mixed for 20 min at room temperature and then stored at 4 °C until further use.

Silver nanoparticles (Ag NPs) with an LSPR at about 407 nm were synthesized according to the Lee and Maisel citrate reduction method.^32^ Briefly, 1.7 mL of a solution of 1% w/v silver nitrate was added to 100 mL of Milli-Q water, and the mix was boiled under vigorous stirring. Once boiling was reached, 2 mL of 1% w/v trisodium citrate was added to the reaction solution, which was stirred and boiled for about 1 h, until the solution changed its color from transparent to gray/silver. After cooling to room temperature, the colloidal solution was stored at 4 °C until further use.

Silver nanoprisms (Ag NPrs) were obtained through a thermal process by following the methods reported in the literature with minor modifications.^33^ In particular, the following reagents were added to a 250 mL flask containing 50 mL of Milli-Q water in the following order: 250 μL of 20 mM silver nitrate, 3 mL of 30 mM trisodium citrate, 3 mL of a solution of 0.7 mM PVP, 120 μL of 30% w/w H_2_O_2_. These were maintained under vigorous stirring at room temperature, when 500 mL of a freshly prepared solution of 100 mM NaBH_4_ was rapidly added to the mix, resulting in an initial pale yellow color. The colloidal solution was stirred in the dark for 2 h until the color changed from yellow to vibrant blue, confirming the change in the shape of the colloidal particles. The solution was stored at 4 °C until further use.

### 2.3 Characterization

A Carl Zeiss Supra scanning electron microscope (SEM) was used to obtain scanning electron microscopy images (accelerating voltages in the range of 5–10 kV). The SEM was connected to an Oxford Instruments X-Max 50 for energy-dispersive X-ray (EDX) spectroscopy, which was recorded at 10 kV.

To obtain nanoparticle SEM images, diluted solutions were deposited on n-doped Si wafers. Instead, prior to SEM imaging of the paper samples, they were coated with a ∼10 nm film of AuPd (90% Au, 10% Pd).

UV–vis spectra were acquired using an Agilent/HP 8453 UV–vis spectrophotometer (190-1100 nm, spectral bandwidth 1 nm).

The polydispersity index (PDI), hydrodynamic diameters, and ζ potential of the nanoparticle aqueous solutions were obtained using a Zetasizer Instrument (Zetasizer Nano-ZS, Malvern Instruments, Malvern Panalytical, U.K.) based on dynamic light scattering (DLS) and electrophoretic light scattering (ELS).

A Horiba XploRA PLUS Raman Microscope equipped with a 70 mW 532 nm laser was utilized for acquiring Raman spectra (1–10% laser power, 10–50× objectives, 10–60 s). The software was used to process the acquired Raman spectra. Origin 2022 was used for data analysis and scientific plotting.

### 2.4 Nanoink Preparation

From each nanoparticle colloidal solution, ink was prepared to write the metallic SERS substrate on paper using a fountain pen. The inks were obtained through a process involving centrifugation and purification of the specific colloidal solution, resulting in a high-concentration nanoparticle solution. Hence, the term “nanoink” will be used in this work. Au NPs were centrifuged at 10 000 rpm for 20 min, while Ag NPs were centrifuged at 13 200 rpm for 30 min at room temperature. For instance, 2 mL of colloidal solutions was centrifuged, and the pellet obtained was washed with 2 mL of Milli-Q water and recentrifuged, finally obtaining around 150 μL, ensuring that no aggregates were formed on the bottom of the centrifuge tube, indicating instability of the NPs. The final nanoinks were kept in the fridge in the dark to preserve their stability since no additives were added to the mixture.

### 2.5 Preparation of the Paper-Based SERS Sensor

The following commercial papers with different textures and weights were tested as potential SERS substrates: (a) Kodak photo paper 180 GSM (Eastman Kodak Company, Rochester, NY, USA), (b) Ilford pearl photo paper 270 GSM (Harman Technology, Ilford Photo, Knutsford, Cheshire, U.K.), (c) Whatman membrane filter mixed cellulose ester (Whatman, Cytiva, Little Chalfont, Buckinghamshire, U.K.), (d) Whatman qualitative filter paper grade 1 (Whatman, Cytiva, Little Chalfont, Buckinghamshire, U.K.), (e) Daler-Rowney acrylic paper 180 GSM (Daler-Rowney Ltd., Bracknell, U.K.); (f) Xerox 90 GSM (Xerox Corporation, Norwalk, Connecticut, USA), (g) Premier stationery white card 160 GSM (Premier Stationery, Stereame, Nenagh, Co. Tipperary, Ireland), and (h) Bristol smooth surface 270 GSM (Strathmore Artists Papers, Appleton, Wisconsin, USA).

To fabricate the SERS sensor, the synthesized nanoinks were used to fill the cartridges of the fountain pens. SERS substrates were generated by drawing squares (about 0.25 cm^2^) on clean paper. A double-layer approach was used to write the nanoink squares, whereby squares were drawn in the horizontal and vertical directions to ensure high coverage of NPs in the analytical area. The paper with the deposited nanoink was left to dry at room temperature before drop-casting a small volume of the analyte onto a square (10 μL). Then, the deposited sample was dried at 50 °C for 30 min prior to Raman analysis. To summarize, Scheme 1 reports the preparation process of the label-free, paper-based SERS sensor.

**Scheme 1:**
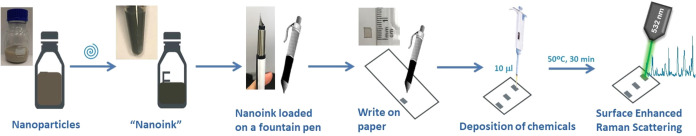
Schematic Representation of the Preparation of the Paper-Based SERS Sensor

To perform SERS analyses, a solution of 0.01 M (4100 ppm) of CV (in ethanol 96%) was used to prepare all further dilutions. Instead, fresh milk was diluted 1:10 in deionized water and spiked with standard solutions of tetracycline hydrochloride at decreasing concentrations.

## 3 Results and Discussion

### 3.1 Characterization of the Metallic Nanoparticles

Figure 1a–d shows the SEM images of all synthesized NPs. The Au NPs, synthesized by citrate reduction of tetrachloroauric­(III) acid, showed spherical homogeneous shape with an average diameter size of 26.9 ± 3.4 nm (Figure 1a). The F-Au NPs were synthesized by a seeding growth approach, through a two-step reduction: (1) a weak reduction at room temperature by citrate (Au^III^ to Au^I^) and (2) a preferential reduction on the gold seed surface by hydroquinone (Au^I^ to Au^0^).^34^ The SEM image (Figures 1b and S1a) showed a flower shape, containing numerous branches (petals). The average diameter dimension was calculated as 88.5 ± 8.4 nm; the variability is mostly due to the different lengths of the petals. The formation of the petals seemed to be related to the random attachment of small Au NPs, which started during the process, on the seed surface, and it did not seem to be dominated by an epitaxial mechanism.^35^ Ag NPs, synthesized through a reduction method of the silver nitrate, showed a quasi-spherical shape (Figure 1c). The average dimension for Ag NPs was 78.9 ± 16.1 nm. While the majority of particles were spherical, other shapes, including ellipsoids and rods, were formed. A simple, one-flask thermal process was used to synthesize Ag NPrs. In this process, the concentration of the reducing agent NaBH_4_ influenced the conversion of small nanoparticles (indicated by the initial pale yellow color of the solution) to NPrs. Triangular NPrs were formed, as shown in the SEM images in Figures 1d and S1b, and tended to arrange themselves in stacks. Ag NPrs had an average edge length of 32.1 ± 7.9 nm, with a more homogeneous thickness (average 6.9 ± 1.3 nm). Insets of Figure 1a–d show photographs of the colloidal solutions obtained, showing intense red (Au NPs), blue (F-Au NPs, Ag NPrs), and gray colorations (Ag NPs). Figure 1e–g shows the UV vis spectra of the synthesized particles. Au NPs showed a UV–vis spectrum characterized by LSPR centered at 527 nm (Figure 1e). The UV–vis spectrum of the F-Au NPs (Figure 1f) presented a broad LSPR centered at 618 nm. The UV–vis spectrum of Ag NPs (Figure 1g) was characterized by a broadband LSPR centered at 407 nm, confirming the heterogeneity in the size of the synthesized particles. Finally, Figure 1h shows the UV–vis spectrum obtained for Ag NPrs, characterized by a main absorption band at 685 nm, corresponding to in-plane dipole plasmon resonance, a weaker band at 465 nm corresponding to the in-plane quadrupolar resonance, and a sharp but small peak at 330 nm related to the out-of-plane quadrupole resonance.^36,37^

**Figure 1:**
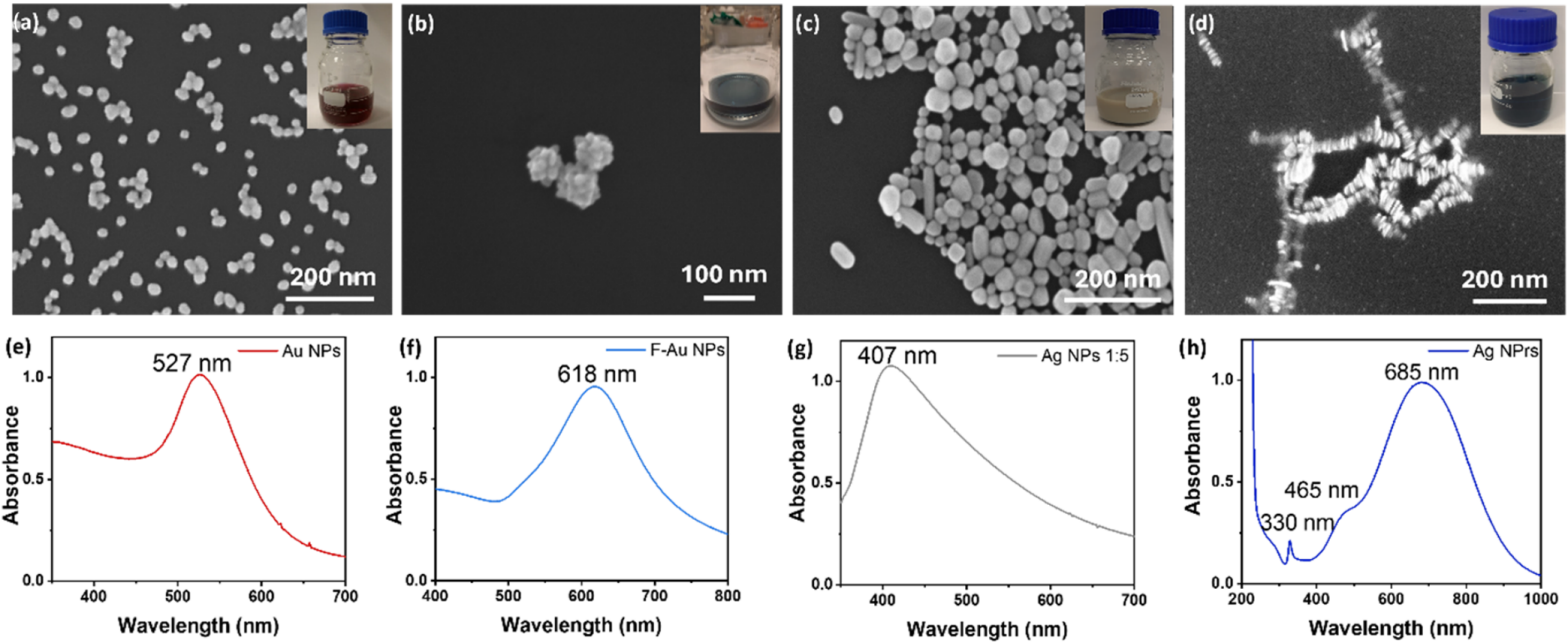
Characterization of the synthesized metallic nanoparticles. SEM images of (a) Au NPs (voltage: 5 kV; magnification: 40k×); (b) F-Au NPs (voltage: 8 kV; magnification: 100k×); (c) Ag NPs (voltage: 8 kV; magnification: 50k×); and (d) Ag NPrs (voltage: 10 kV; magnification: 80k×). Insets: digital pictures of the NP solutions. (e–h) UV–vis spectra of (a)–(d).

Together with SEM and UV–vis spectroscopy, the nanoparticles were characterized using DLS and ELS, carrying out measurements at 25 °C after 120 s of equilibration time between each sample. The data are reported in Table S1, obtained by analyzing 3 samples per material, and, for each sample, 3 measurements (12 runs each) were recorded. The solutions were analyzed as freshly prepared and without any pretreatment, except for the Ag NP solution, which was diluted 1:5 prior to measurement.

### 3.2 Nanoink Preparation

To obtain a SERS substrate, it was necessary to prepare an “ink” for each synthesized nanoparticle solution. The use of an ink was key to obtain a controlled deposition of NPs on the paper, avoiding the spreading of the solution during pen writing, and to obtain a high concentration of nanoparticles on the paper, leading to SERS functionality. Usually, an ink is a liquid, semiliquid, or solid composed of different components: a colorant (dye or pigment), a vehicle (water or organic solvent), and other additives (resins, polymers, stabilizers, etc.).^38^ In this work, the nanoink was a liquid composed of highly concentrated nanoparticles, as a colorant, in an aqueous vehicle. No other additives were included in the mixture, except for stabilizing molecules present on the surface of the NPs. The concentration of the initial NP solution was increased through a series of centrifugations, whereby it was ensured that no aggregates were formed at the bottom of the centrifuge tube, indicating the instability of the NPs. Through centrifugation, the concentration (optical density, OD) of all the colloidal solutions increased, as shown in Table 1. In general, an increase of about 14 times in concentration was obtained, with the exception of spherical Au NPs, which could be concentrated 42 times, probably owing to their spherical shape and size homogeneity, which allowed tight compacting. UV–vis spectra were recorded for all nanoinks and are reported in Figure S2.

**Table 1:** LSPR Maximum and Optical Density for All the Nanoparticles Synthesized, before (As-Synthesized) and after the Centrifugation Process (Nanoinks)

material	wavelength solution (λ, nm)	optical density (OD)	wavelength nanoink (λ, nm)	optical density nanoink (OD)
Au NPs	527	1	527	42
F-Au NPs	618	1	616	14
Ag NPs	407	5	407	68
Ag NPrs	685, 465, 330	1	625, 330	14

### 3.3 Evaluation of Substrates for the Paper-Based SERS Sensor

Considering the characteristics of the chosen technique (SERS) and the objective of developing a sensor using a flexible, environmentally friendly, and low-cost substrate, paper was selected as the substrate. To select the optimal paper for this purpose, eight types of paper were investigated, focusing on the ease of pen writing and on their spectral response in the analyte spectral region of interest (300–1800 cm^–1^). Figure 2 displays microscope images of the different paper substrates analyzed, together with digital pictures of the squares written with a fountain pen loaded with Au NP nanoink. Figure 2 also shows the blank SERS response of the written papers, which must be minimal to avoid interference during analyte detection. The fluorescence response was minimized by adjusting the laser power and acquisition time. Specifically, Figure 2a,b shows data related to two photographic papers, Kodak photo paper 180 GSM and Ilford pearl photo paper 270 GSM. Both papers presented a smooth surface, which made pen writing easy and produced smooth colored squares. However, both papers had a glossy texture, probably due to the use of additives, such as resins, minerals, or optical brightening agents, added during the production process.^39,40^ Therefore, their SERS spectral response (insets) was characterized by intense peaks in the fingerprint region, which was the reason why these two substrates were not considered suitable for further use. Figure 2c,d displays the results obtained for two types of filter paper: Whatman membrane filter mixed with cellulose ester, and Whatman qualitative filter paper grade 1. While it was easy to write on the former filter paper, it was not easy to write on the latter since the nanoink spread unevenly, as observed in the written square shown in the inset of Figure 2d. Both Whatman papers were characterized by SERS spectra with no peaks in the region of interest (see the inset images). However, these two substrates were discarded because, upon deposition of an aqueous solution mimicking the deposition of analytes, they wrinkled with the solution spreading beyond the nanoink written area. Figure 2e shows the data related to the Daler-Rowney acrylic paper 180 GSM. This substrate was not suitable, as the writing was not smooth, and the SERS spectrum presented some interfering response in the region of interest. Finally, Figure 2f–h shows the characterization of three printer papers with different surface textures and weights: Xerox 90 GSM, Premier stationery white card 160 GSM, and Bristol smooth surface 270 GSM. These printing papers were considered suitable substrates as fountain pen writing was easy and, mimicking the sample addition, the aqueous solution was confined to the area of interest, owing to their hydrophobicity. Also, the blank SERS spectra for these papers did not show characteristic peaks or high noise in the region of interest. Among the three analyzed printer papers, Bristol was selected for further use, being the smoothest (easy nanoink flow) and heaviest (mechanically robust) of all, while also maintaining good flexibility. A summary of the characteristics of all of the analyzed paper substrates is reported in Table S2.

**Figure 2:**
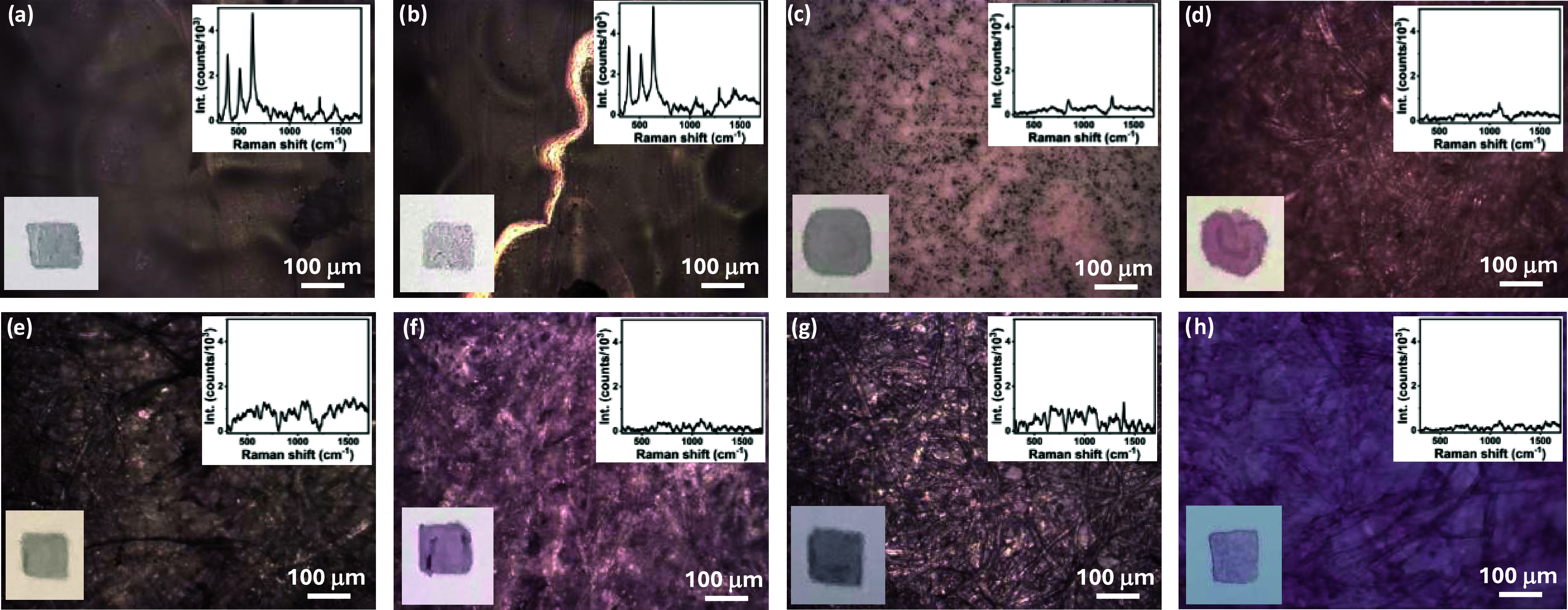
Different paper materials were studied to find the best substrate for our paper-based SERS sensor. Microscope images (10× objective) are shown for: (a) Kodak photo paper 180 GSM; (b) Ilford photo paper 270 GSM; (c) Whatman mixed cellulose ester membrane filter; (d) Whatman qualitative filter paper, grade 1; (e) acrylic paper 180 GSM; (f) Xerox Printer paper 90 GSM; (g) premier stationery white card 160 GSM; and (h) Bristol paper 270 GSM. For all the materials, are also reported, as insets, the SERS spectra obtained (532 nm laser, 10× objective, laser power 0.7 mW, 40–60 s acquisition) and the digital photographs of drawn squares with the Au NP nanoink.

Prior to the use of Bristol paper as a SERS substrate, the stability of the substrate written with nanoinks was tested by scotch tape and water stability tests (Figure S3a–c), further showing suitability for SERS diagnostic applications.

### 3.4 Characterization and Optimization of the Paper-Based SERS Sensor

Considering that the SERS effect is stronger in the gaps between plasmon-coupled metal nanoparticles,^41^ to increase the probability of so-called “hotspots” formation, the nanoink was deposited on the substrate using a double-layer writing strategy. Specifically, the first layer was written by drawing vertical lanes into a square shape. Once this layer dried, a second layer was written, drawing horizontal lines on top of the vertical features and ensuring that all empty areas were covered. In Figure S4, it is possible to observe the visual difference between a single-layer and a double-layer written nanoink, noticing an intensification of the nanoink color intensity. At the same time, Figure 3a shows why the presence of a hotspot can make a difference in residual detection. In fact, Figure 3a displays the actual enhancement obtained by analyzing 10^–10^ M CV in the presence (red line) vs absence (black line) of a hotspot (the intensity of the peak at 1175 cm^–1^, often used as reference for CV, was 4.5 times more intense in the presence of a hotspot).

**Figure 3:**
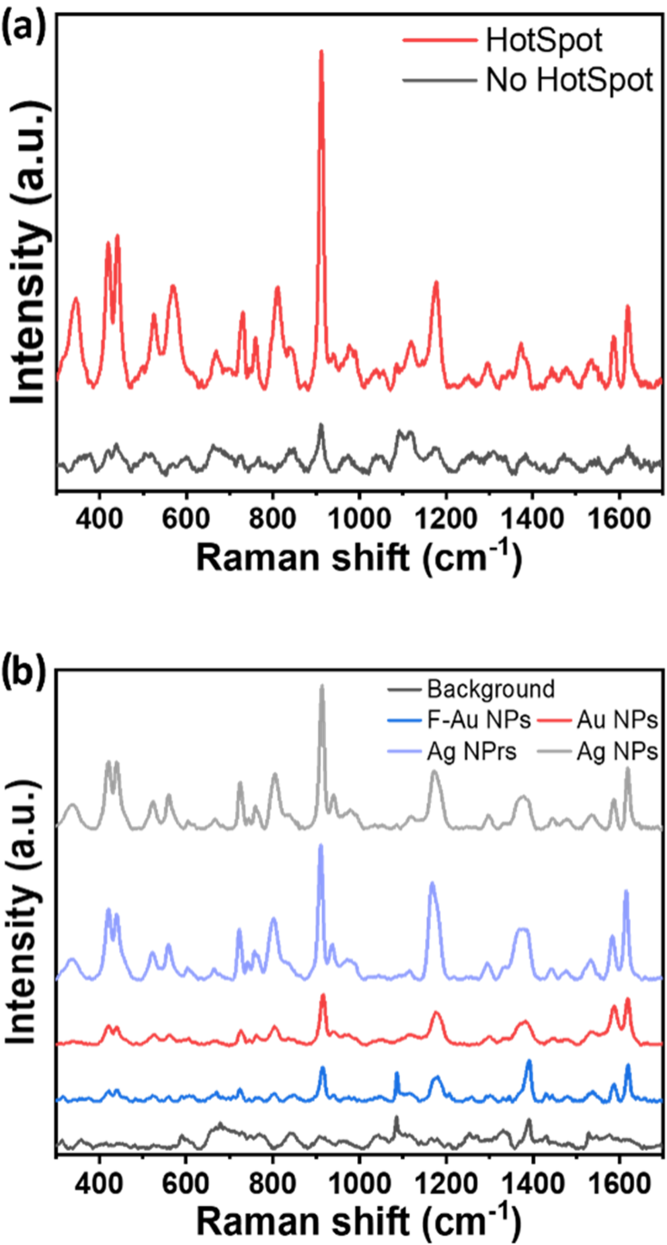
(a) SERS spectra recorded in a hotspot (red line) vs in a no-hotspot area (black line) of 10^–10^ M crystal violet deposited on the nanoink square (laser 532 nm, objective 50×, laser power 0.25%, acquisition time 80 s). (b) SERS spectra of 10^–4^ M crystal violet (CV) on different nanoinks (laser 532 nm, objective 50×, laser power 0.175–0.35 mV, 30–80 s); from the bottom to the top: background spectra (black); F-Au NPs (blue); Au NPs (red); Ag NPrs (indigo); Ag NPs (gray).

To further optimize the sensitivity of the sensor, four nanoinks made from the 4 synthesized metallic nanoparticles were investigated with the Raman model molecule CV to investigate their SERS effect. To this end, a 10^–4^ M CV solution was used to compare the SERS response of the different nanoinks. Figure 3b displays the SERS spectra obtained with a laser excitation of 532 nm, showing that the Ag nanoinks (NPs and NPrs) produced greater signal enhancement than the other nanoinks. Therefore, the two Ag nanoinks were further investigated at different CV concentrations.

Prior to starting the analysis, the density of NPs deposited on the paper substrate following pen writing was investigated by SEM. Figure S5 shows the images of the chosen substrate before and after pen writing, making evident the differentiation between the two; in fact, in Figure S5b, the fibers are filled with nanoparticles. In Figure S5a, some structures are also visible, which, according to the analysis through SEM and energy-dispersive X-ray spectroscopy (SEM/EDX) reported in Figure S6, should be calcium carbonate (CaCO_3_), often used as a filler during paper production.

### 3.5 Crystal Violet Case Study: Ag NPs vs Ag NPrs

CV was chosen as a Raman probe owing to its spectroscopic properties and fingerprint spectrum as well as its use, together with its derivatives, in many industrial applications and as a veterinary drug for the treatment of ornamental fish infections. CV use is actually illegal and strictly controlled in fish destined for human consume since its residues are toxic for humans once absorbed by the animal.^42^ Since laborious techniques (mostly liquid chromatography) are usually used for detecting CV and its derivatives, there is a great interest in finding alternative detection methodologies to simplify and accelerate CV analysis.^43^ The UV–vis spectroscopy characterization, the Raman characterization of the powder, and the characteristic peaks are reported in Figure S7a,b and Table S3, respectively. Among different techniques, SERS has been used for determining the presence of CV in different platforms using different metallic nanostructures.^44–46^

Figure 4 shows the SERS spectra obtained with both Ag nanoinks (Ag NPs (a), Ag NPrs (b)) at different concentrations of CV. Specifically, 10^–8^ M CV (4.1 × 10^–3^ ppm = 4.1 ppb) was the lowest concentration detectable with the Ag NP nanoink, where all of the characteristic peaks of the molecule were still visible. Ag NPrs nanoinks demonstrated a higher sensitivity, and detected CV at a concentration of 10^–12^ M (4.1 × 10^–7^ ppm = 0.00041 ppb).

**Figure 4:**
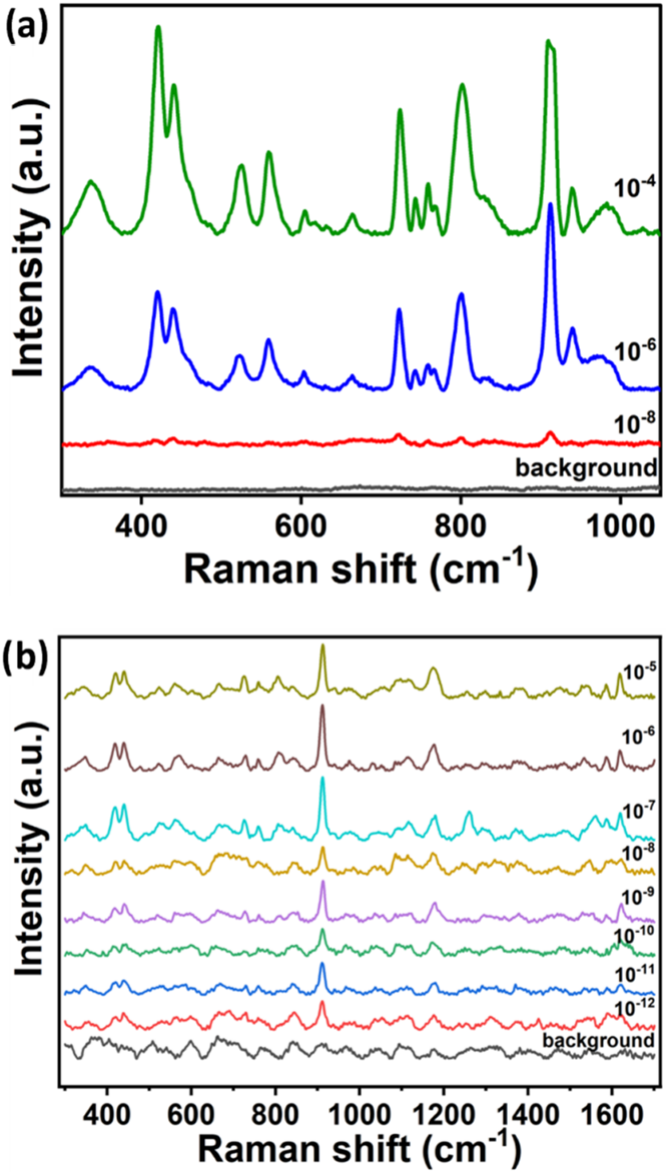
(a) SERS spectra of CV at three different concentrations (10^–4^, 10^–6^, 10^–8^ M in EtOH) obtained on the Ag NP nanoink sensors in comparison with the background spectrum. All spectra were collected at 532 nm, objective of 10×, an acquisition time of 30 s, and a laser power of 0.7 mV. (b) SERS spectra of CV in EtOH at diluting concentrations (from 10^–5^ to 10^–12^ M) obtained on the Ag NPrs nanoink sensors. All spectra were collected at 532 nm, objective of 50×, an acquisition time of 80 s, and a laser power of 0.175 mV.

Considering the possibility of detecting 8 concentrations using the paper-based sensor with Ag NPrs nanoink, a calibration curve was built for the detection of CV. In particular, five different spectra per concentration were collected at different points on the square of the paper-based sensor, and the intensities of the characteristic peak at 1175 cm^–1^ were analyzed in order to obtain the mean intensity and its relative error. Decreasing the concentration of CV also decreased the signal of the diagnostic peak; in particular, the curve in Figure S8 was obtained through a nonlinear regression analysis of the data using a four-parameter logistic equation, with a correlation coefficient *R* ^2^ = 0.978.

Both the Ag nanoinks were proven to be suitable for SERS investigations. However, stability analyses conducted on both suspensions showed that Ag NPrs were unstable after 21 days (Figure S9a), showing a shift in the wavelength of maximum absorbance, while Ag NPs remained stable. Furthermore, the synthesis of Ag NPrs showed poor reproducibility, with batch-to-batch variability of the maximum absorbance wavelength, as observed in the UV–vis spectra shown in Figure S9b and Table S4. This reproducibility problem was not observed for the quasi-spherical Ag NPs, which were chosen for the analysis of antibiotic residues in milk samples.

### 3.6 Tetracycline Detection

Tetracycline hydrochloride is one of the most commonly used antibiotics in veterinary medicine. To find a fast, simple, and low-cost method to detect it, it is necessary to monitor its residual presence in foods destined for consumers. The Raman spectrum of tetracycline powder is shown in Figure S10a, while Table S5 shows Raman assignments, in agreement with the literature, with some minor shifts due to the laser used.^47^ Figure S10b shows the SERS spectrum of tetracycline solution deposited on an Ag NP nanoink square, where the enhancement in tetracycline Raman signals can be observed, with a minor shift for some peaks compared to the Raman spectrum. For quantitative analysis of tetracycline in DI water, antibiotic solutions at different concentrations (1000, 500, 100, 50, 20, 10, 5, 2, 1, 0 ppm) were prepared. 10 μL of each solution was deposited on different paper-based SERS sensors written with Ag NP nanoinks and measured under 532 nm laser excitation. The analysis was carried out through a mapping process, choosing nine different points across the nanoink square, and the results obtained for this analysis are shown in Figure S11.

For milk analysis of tetracycline residues, it is necessary to consider the complexity of the matrix. It is composed of about 87% water, ∼3% protein, ∼4% fat, ∼5% lactose, and ∼1% vitamins and minerals. Raman spectroscopy has been recently used to investigate milk quality and composition since fats, proteins, and lactose (milk solids) are all Raman-active molecules. In fact, in the literature, it is reported how the change in milk solids could affect the Raman signals.^48^ Instead of using milk with a lower amount of fats, for instance, light milk (usually containing around 1 g of fat in 100 mL) or fat free milk (containing around 0.1 g of fat in 100 mL), we analyzed whole milk and diluted it to diminish all solid contributions on the SERS analysis.

Specifically, milk was diluted 1:10 in water and spiked with different concentrations of the antibiotic. 10 μL of each sample (500, 250, 100, 50, 25, 10, 5, 2.5, 0 ppm tetracycline in milk) was deposited on the paper-based sensors and dried before SERS analysis. The peaks obtained are in accordance with those reported in the literature, slightly shifted due to the interactions of the antibiotic with the milk matrix.^2^

Figure 5a,b shows representative SERS spectra obtained at different concentrations of tetracycline in milk, with a zoom-in at lower concentrations (from 25 to 0 ppm). The SERS analysis was carried out using 4 different points on the Ag NP nanoink square written with a pen-on-paper approach, using the following parameters: laser 532 nm, objective 10×, laser power 0.7 mW, and acquisition time 30 s. As observed for the molecular probe, decreasing the concentration of the antibiotic reduced the intensity of the SERS peaks. Specifically, as shown in Figure 5c, there was a logarithmic correlation between the intensity of the chosen peak of interest for tetracycline (1617 cm^–1^) and the concentration of the antibiotic (correlation coefficient *R* ^2^ = 0.960). At the same time, a linear dependence of the SERS intensity of the peak at 1617 cm^–1^ vs the log of the concentration was found for the range 250–2.5 ppm, with a correlation coefficient *R* ^2^ = 0.906 (Figure 5d). The limit of detection (LOD) and the limit of quantification (LOQ) of our method were calculated as (3 × σ)/*s* and (10 × σ)/*s*, respectively, with σ as the standard deviation of 4 blank measurements and *s* as the slope of the calibration curve (Figure 5d). The LOD calculated is 0.07 ppm, while the LOQ is 0.23 ppm. Moreover, for the lowest measurable concentration (2.5 ppm), the signal-to-noise ratio (S/N) was also calculated, obtaining a value higher than 3, thereby meeting the LOD criteria (S/N = 20.15). For this calculation, we used the following formula:

$$S/N=\frac{p\mathrm{eak}\;i\mathrm{ntensity}\;\left(S\right)}{s\mathrm{tandard}\;d\mathrm{eviation of}\;n\mathrm{oise}\;\left(\sigma_{noise}\right)},\mathrm{using for both terms the average of}\;4\;\mathrm{measurements}$$

**Figure 5:**
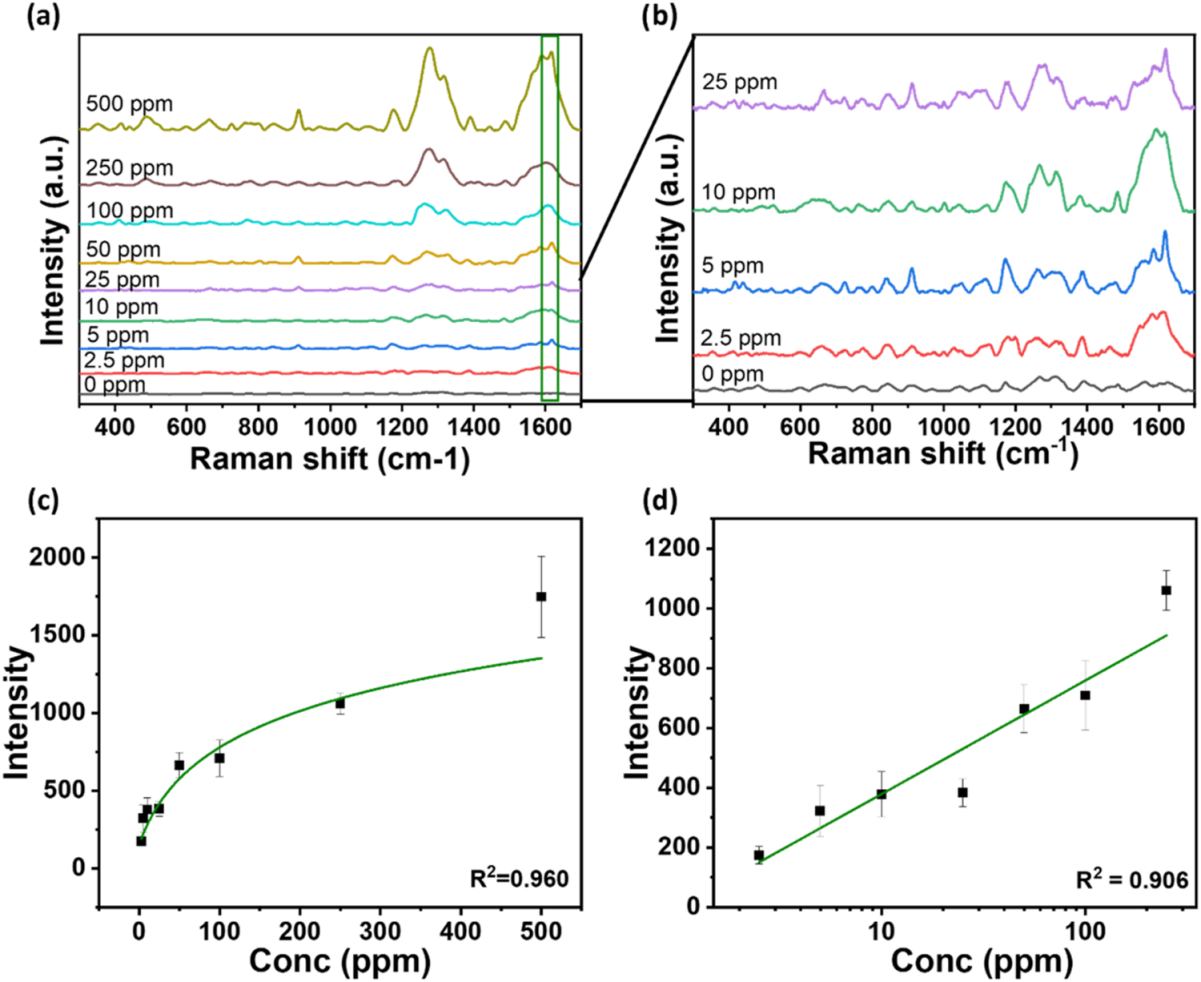
(a) SERS analysis of different concentrations (0–500 ppm) of tetracycline in milk. (b) Zoom-in of the 5 lowest concentrations (0–25 ppm) in milk. (c) SERS intensity 1620 cm^–1^ vs concentration of tetracycline. (d) Calibration curve obtained through linear fitting of the SERS intensity at 1620 cm^–1^ vs log of tetracycline concentration. The error bars refer to 4 measurements.

A comparison between our paper-based SERS sensor and other detection methods reported in the literature for tetracycline in milk is presented in Table S6.

## 4 Conclusions

In this work, the combination of Bristol paper and Ag NP nanoinks has allowed us to obtain simple, low-cost fabrication label-free paper-based SERS sensors. The Ag NP nanoinks were readily prepared and placed on paper through a simple, DIY pen-on-paper process. The mechanical and diagnostic properties of the SERS sensor were successfully assessed by using CV as a SERS probe. The ease of fabrication and the demonstrated capability of the sensor to detect low concentrations of tetracycline in a complex matrix such as milk point to the possible use of such disposable label-free SERS sensors in third-world countries or locations with limited access to trained personnel and expensive analytical instruments. Moreover, this opens the possibility of detecting different contaminants in food matrices as well as different analytes in diverse complex matrices, possibly in a point-of-site scenario, in combination with portable Raman instrumentation.

## Supplementary Material



## Notes

SEM images of F-Au NPs and Ag NPrs; PDI, hydrodynamic diameters, and ζ potentials of the diverse synthesized nanoparticle solutions; characterization of nanoinks (UV–vis spectroscopy); evaluation and characterization of paper substrates (mechanical stability, deposition strategy, SEM images, and EDX); characterization of CV (UV–vis and Raman spectroscopy, calibration curve obtained); stability and reproducibility of Ag NPrs; and characterization of tetracycline (Raman vs SERS) and analysis of tetracycline in water (PDF)

## Abbreviations

Ag NPrs: silver nanoprisms
Ag NPs: silver nanoparticles
Au NPs: gold nanoparticles
CE: chemical enhancement
CV: crystal violet
DIY: do-it-yourself
EDX: energy-dispersive X-ray spectroscopy
ELISA: enzyme-linked immunosorbent assay
EM: electromagnetic enhancement
F-Au NPs: flower-shape gold nanoparticles
LOD: limit of detection
LOQ: limit of quantification
LSPR: localized surface plasmonic resonance
MRLs: maximum residue limits
OD: optical density
SEM: scanning electron microscopy
SERS: surface-enhanced Raman spectroscopy
