# Supplementary material for: Pen-on-Paper Label-Free Surface-Enhanced Raman Scattering (SERS) Detection of Tetracycline in Milk
Source: ACS Omega. 2025 Jun 6;10(23):24710–9. doi: 10.1021/acsomega.5c01637 (PMC12177620; doi:10.1021/acsomega.5c01637)
Supplement: Supplementary file 1 [file ao5c01637_si_001.pdf]

SUPPORTING INFORMATION

Pen on Paper label-free Surface Enhanced Raman Scattering (SERS) Detection of Tetracycline in Milk

Alida Russo [a], Martina Piletti [a], Aidan J. Quinn [a], Daniela Iacopino[a*]

[a] Tyndall National Institute, University College Cork, Lee Maltings Complex, Dyke Parade, T12R5CP, Cork, Ireland

*Corresponding author

Characterization of metallic nanoparticles

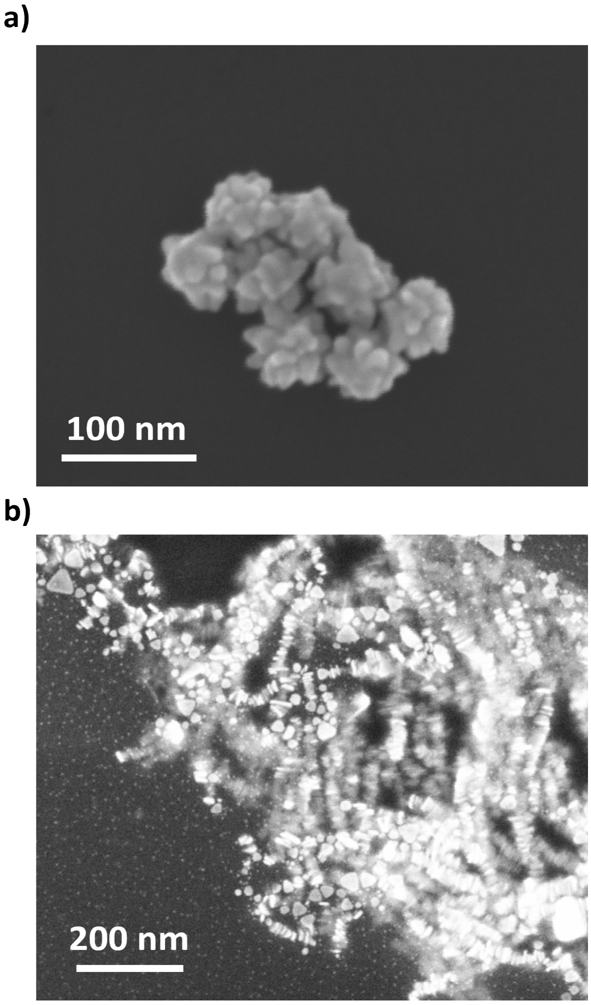

Figure S1: SEM images for a) F-Au NPs (voltage: 8 kV; magnification: 125 KX); b) Ag NPrs (voltage: 10 kV; magnification: 60 KX).

Table S1: Comparison of Polydispersity index (PDI), hydrodynamic diameters and zeta potentials between the different plasmonic nanoparticles.

Material	PDI	Peak 1 (d.nm)	Peak 2 (d.nm)	Peak 3 (d.nm)	Z-Average	Z-Potential (mV)
**Au NPs**	0.63 ± 0.03	43.3 ± 4.4	2.12 ± 0.1	-	20.3 ± 0.8	-33.3 ± 3.5
**F-Au NPs**	0.16 ± 0.02	83.4 ± 1.6	-	-	69.6 ± 1.9	-26.4 ± 2.2
**Ag NPs**	0.26 ± 0.01	93.0 ± 1.5	-	-	64.6 ± 0.4	-33.2 ± 1.9
**Ag NPrs (1),** $\lambda_{max =}$ 838 nm	0.55 ± 0.03	44.1 ± 6.3	6.33 ± 7.5	1.2 ± 0.8	24.0 ± 1.8	-22.02 ± 1.8
**Ag NPrs (2),** $\lambda_{max =}$ 733 nm	0.59 ± 0.02	38.9 ± 2.8	1.5 ± 0.2	1.1 ± 1.2	19.6 ± 1.1	-21.1 ± 1.1

UV-vis characterization of nanoinks

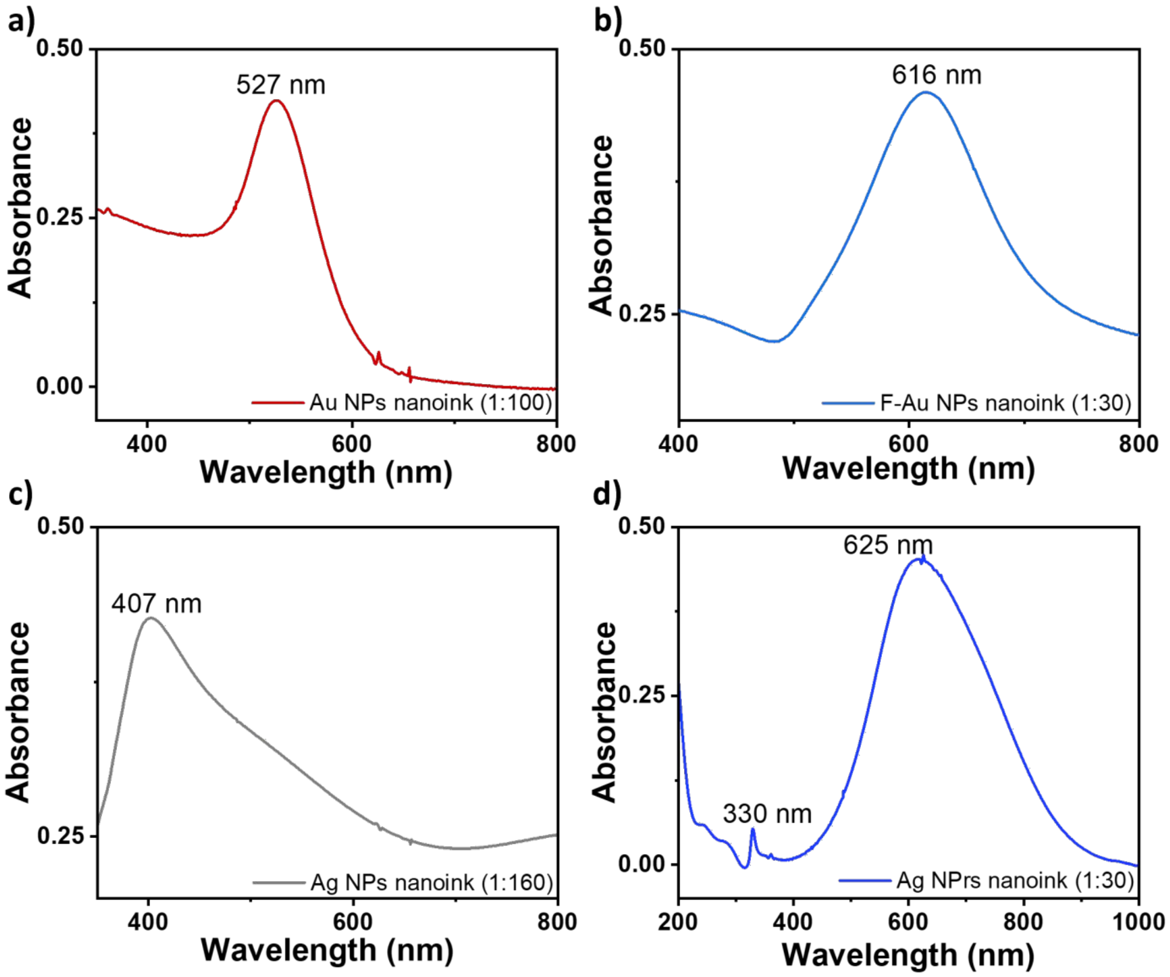

Figure S2: UV-vis spectra of the diluted nanoinks, prepared from the colloidal solutions synthesized: a) Au NPs (dilution 1:100); b) F-Au NPs (dilution 1:30); c) Ag NPs (dilution 1:160); d) Ag NPrs (dilution 1:30).

Evaluation of substrates for the paper-based SERS sensor

Table S2: Summary on the paper substrates' characteristics studied for the development of a paper-based SERS sensor.

**Paper substrate**	**Ease of writing on substrate**	**Presence of Raman peaks in the fingerprint region**
Kodak photo paper (180 GSM)	Yes	Yes
Ilford pearl photo paper (270 GSM)	Yes	Yes
Whatman membrane filters mixed cellulose ester	Yes	No
Whatman qualitative filter paper, Grade 1	No	No
Daler-Rowney Acrylic paper (180 GSM)	No	No
Xerox Printer paper (90 GSM)	Yes	No
Premier Stationery white card (160 GSM)	Yes	No
**Bristol paper (270 GSM)**	**Yes**	**No**

Mechanical stability of the paper substrate

Adhesion of nanoink to the paper was tested to assess the mechanical stability of the SERS substrates. Scotch tape and water stability tests were conducted.[1] For the scotch test (Figure S2a-c), a nanoink square was written on the Bristol paper and was let dry to obtain a SERS sensor, after which a piece of scotch tape was pressed on it and pulled off. As visible, the nanoink did not detach from the substrate, meaning that the particles were strongly attached to the paper through electrostatic and chemical interactions. For the water stability test (Figure S2d-f), a dried written SERS square was immersed in DI water for 24 hours and, subsequently, extracted and left to dry at room temperature. No particles were visible in the water after removing the sample and no visible detachment from the paper was observed. As for the previous test, the substrate resulted stable enough after water soaking.

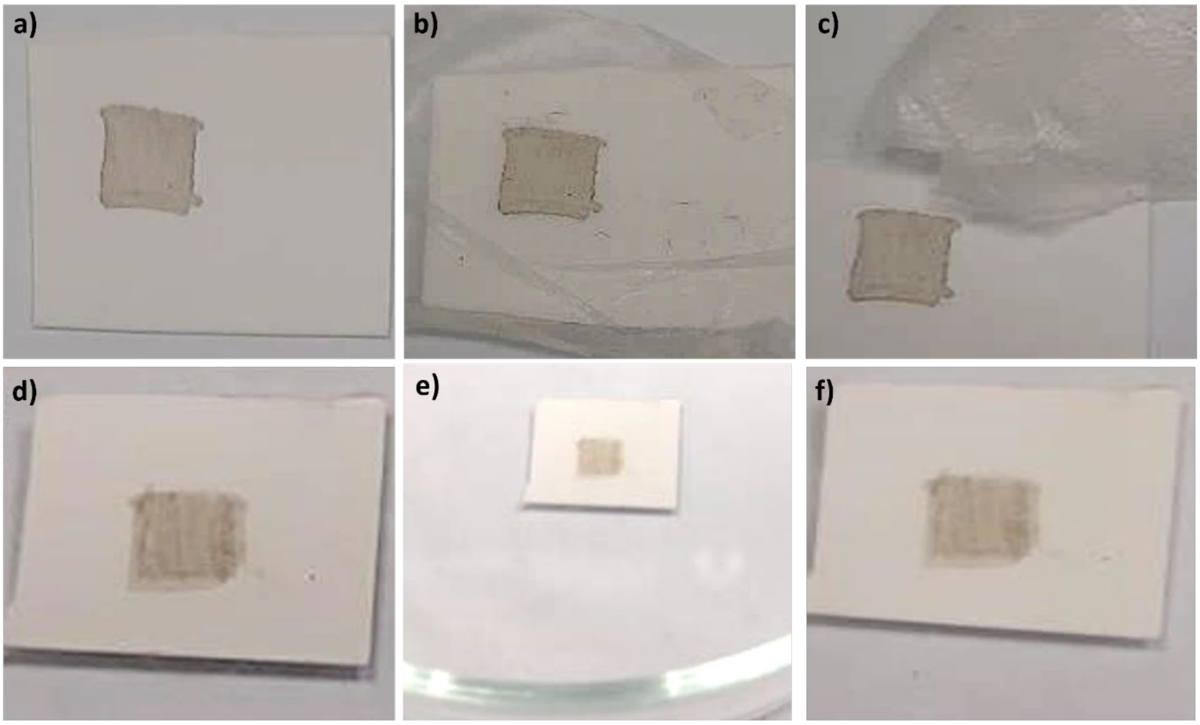

Figure S3: Digital pictures of: a-c) scotch tape test and d-f) water resistance test performed on Bristol paper with pen written Au NPs nanoinks.

Characterization and optimization of the paper-based SERS sensor

In order to obtain a higher number of deposited nanoparticles, hence increasing the probability on finding ‘hotspots’ along the written square, the nanoink was deposited on the paper substrate using a double-layer writing strategy. Figure S3 shows both single-layer and double-layer approach deposition.

Instead, Figure S4 displays the SEM images of a) blank paper substrate and b) nanoink double-layer deposited paper.

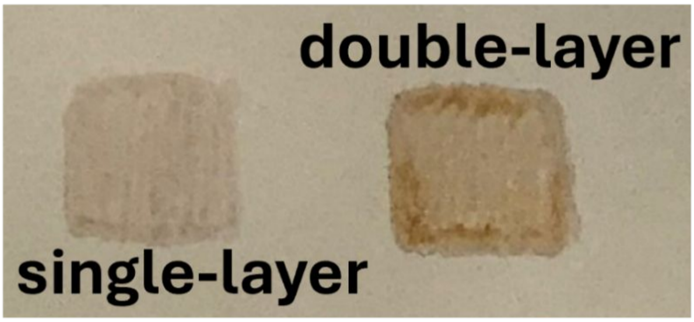

Figure S4: Digital pictures of the fountain pen written squares with AgNPrs nanoink with both a single-layer and double-layer strategy.

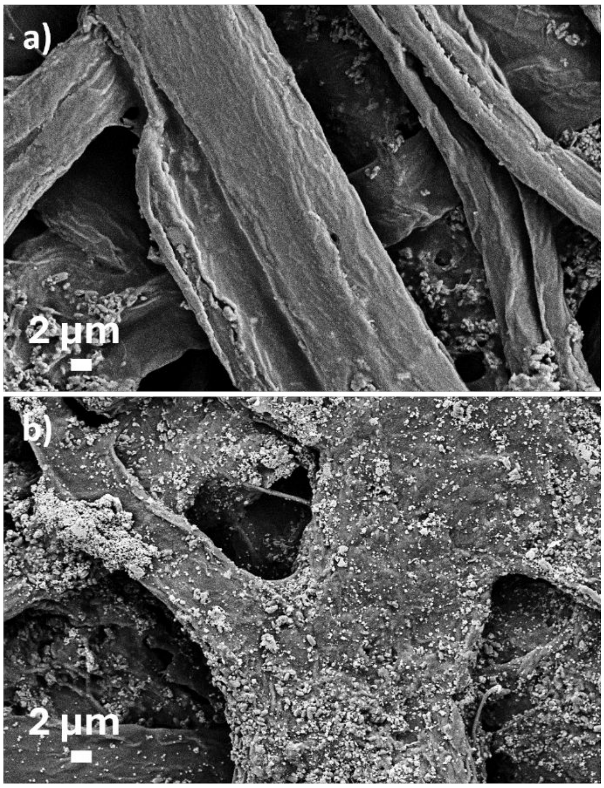

Figure S5: Scanning electron microscope images (voltage: 5kV, magnification: 2 KX) of Bristol paper 270 GSM (a) and of the fountain pen written square with the Ag NPs nanoink on same substrate (b).

Calcium carbonate ($CaCO_3$) is one of the most common filler used in papermaking[2,3] to give overtime stability and structure to the paper, especially using alkaline treatments, and this particular paper specify to be acid free, making stronger the hypothesis that it may contain it, as shown in Figure S5.

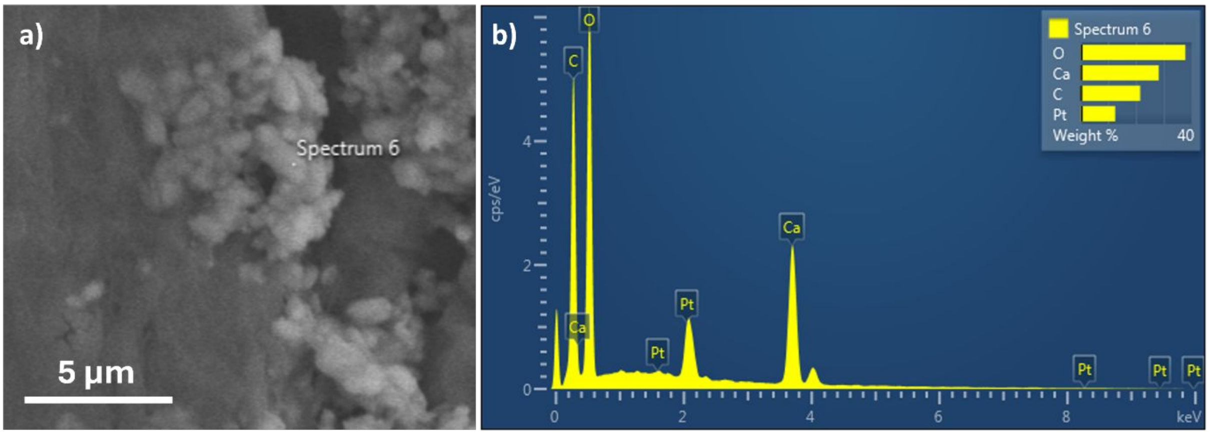

Figure S6: a) SEM image of Bristol paper 270 GSM at 10 KV, focusing on the structures presented in the fibres and b) their EDX spectrum where is visible the presence of O, Ca and C connected to the use of calcium carbonate ($CaCO_3$) as filler in the papermaking industry.

Crystal violet case study: AgNPs vs Ag NPrs

Crystal violet (CV) was chosen as molecular probe for studying the SERS capability of our paper-based sensor. Before proceeding on its study, it was characterized through UV-vis and Raman spectroscopy.

In Figure S6a, it is possible to observe the UV-vis spectrum of CV in ethanol. It presents a maximum absorption at 589 nm, with a shoulder at 550 nm, but as well some peaks at 250 nm, 280 nm and 304 nm.[4]

Figure 6b shows the Raman spectrum of CV powder and its assignments are reported in Table S2, as stated in literature.[5,6]

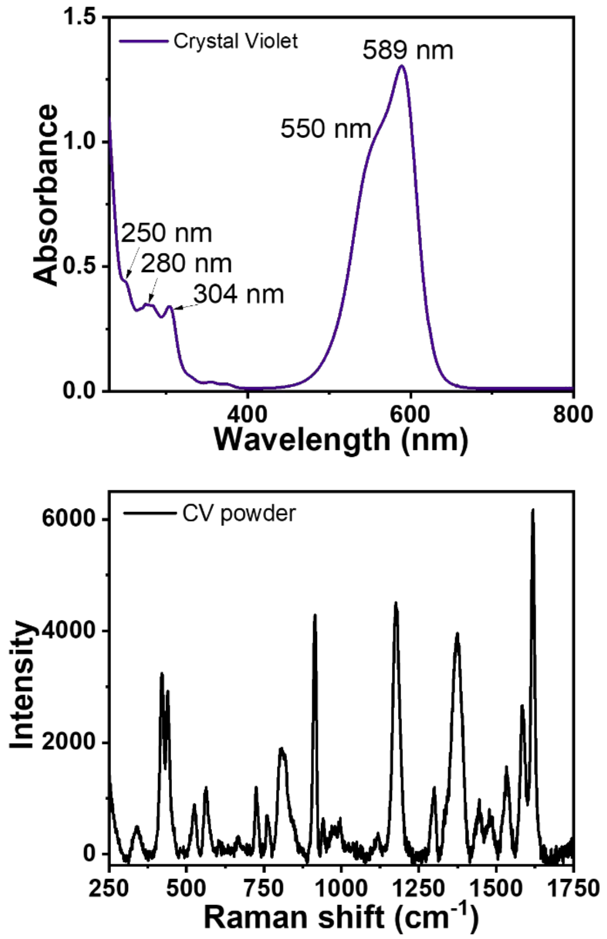

Figure S7: a) UV-vis spectrum of crystal violet 10^{-5} M in EtOH; b) Raman spectrum of crystal violet powder (laser 532 nm, objective 10X, acquisition 80 s, laser power 0.35 mW).

Table S3: Assignments of Raman peaks of crystal violet.

Peak (cm^{-1})	Assignment
**336**	C-C_{center}-C bending out of plane
**421**	C-N-C bending in plane
**439**	C-C ring bending out of plane
**525**	C-N-C antisymmetric bending
**562**	C-C-C deformation out of plane
**725**	C-N-C symmetric stretching
**762**	C-C_{center}-C symmetric stretching
**810**	C-H bending
**914-940**	C-C ring stretching
**1177**	C-H ring bending in plane
**1373**	C-N, C_{center}-C stretching
**1535**	C_{ring}-N antisymmetric stretching
**1585**	C-C ring antisymmetric stretching in plane
**1619**	C-C ring antisymmetric stretching in plane

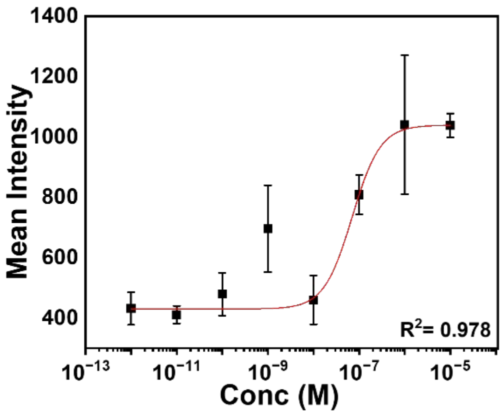

Figure S8: Quantitative analysis of CV using the handwritten paper-based sensor with Ag NPrs nanoink: calibration curve obtained considering the mean intensity of the reference peak for CV (1175 cm^{-1}) considering 5 spectra on different points of the square.

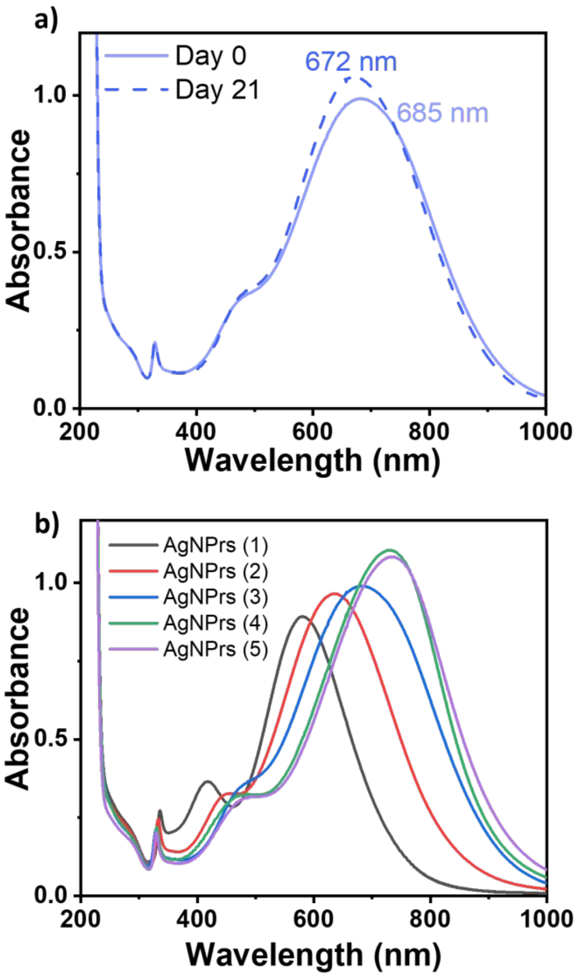

Figure S9: a) UV-vis spectra of the same batch of synthesized Ag NPrs at day 0 (solid line) and day 21 (dashed line); b) UV-vis spectra of 5 different synthesis of Au NPrs under the same conditions (their λ_{max} and OD are reported in Table S2).

Table S4: Wavelengths of the maximum absorption peak and optical densities (ODs) of 5 batches of AgNPrs synthesis carried out at the same conditions.

Synthesis	λ_{max} (nm)	OD
**Batch 1** (black line)	580	0.89
**Batch 2** (red line)	635	0.97
**Batch 3** (blue line)	685	0.99
**Batch 4** (green line)	730	1.10
**Batch 5** (purple line)	735	1.08

Tetracycline detection

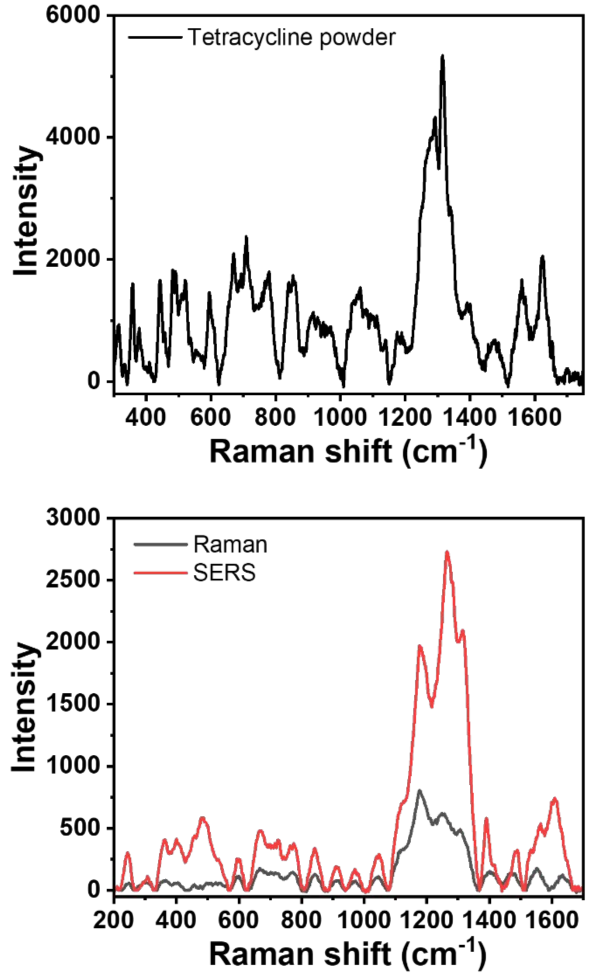

Figure S10: a) Raman spectrum of tetracycline hydrochloride powder (laser 532 nm, objective 10X, acquisition 30 s, laser power 0.7 mW). b) Comparison between a Raman spectrum of tetracycline solution 500 ppm on paper without any metallic ink (black line) and a SERS spectrum of the same solution on Ag NPs ink on paper (red line) (532 nm laser, objective 10X, 10 s, laser power 0.125 mW).

Table S5: Assignments of Raman peaks for tetracycline hydrochloride.

Peak (cm-1)	Assignment
**441**	C-N bending
**482-493**	C-N bending
**520**	Amid-ONH bending
**670-708-778**	Ring breathing, C-H rocking
**933**	Ring deformation, C-H rocking
**1160-1184**	C-C stretching, C-H rocking
**1293**	Breathing ring, C-C stretching, C-O stretching
**1314-1395**	C-H rocking
**1473**	C-O-H bending, C-H rocking
**1561**	N-H scissoring
**1623**	C-C symmetric stretching, C-O-H bending

Figure S10 shows a) the average SERS spectra at different concentrations of tetracycline in water, b) a zoom-in at the lower concentrations, c) and d) curves obtained analyzing the correlation between two characteristic peaks of the SERS spectra of tetracycline (1274 cm^{-1} and 1620 cm^{-1}, respectively) and the antibiotic concentration in the sample. For both, it was found a decrease of the peak intensity reducing the concentration of antibiotic and a sigmoidal dependence of the SERS intensity *vs.* the logarithm of tetracycline concentration. The two curves were obtained through a four-parameter logistic regression, both attaining a correlation coefficient $R^2 > 0.920$.

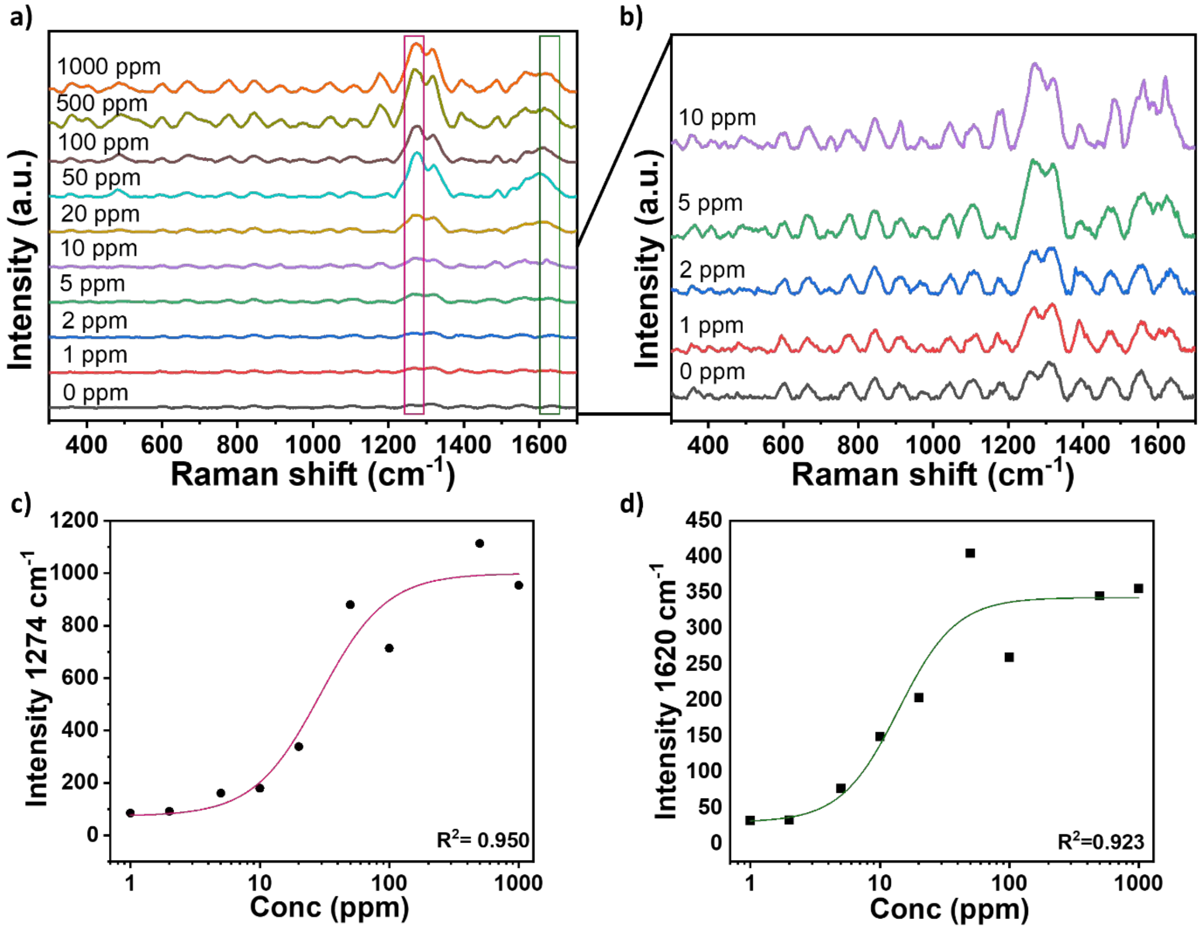

Figure S11: a) SERS analysis of different concentrations (0 - 1000 ppm) of tetracycline antibiotic in water. b) Zoom-in of the 5 lowest concentrations of tetracycline (0 – 10 ppm) in water. c) Calibration curve obtained analyzing one of the fingerprint peak for tetracycline in water: 1274 cm^{-1}. d) Calibration curve obtained analyzing one of the fingerprint peak for tetracycline in water: 1620 cm^{-1}. Both curves

do not show error bars because they are the analysis of average spectra obtained through mapping procedure.

Literature comparison

Table S6: Literature comparison between the proposed method and other methodologies for detection of tetracycline antibiotic in milk.

Method	Cost	Time of analysis	LOD	Reference
**HPLC-UV**	High cost	20 minutes	0.00023 mg/mL	[7]
**Electrochemical immunosensor**	Low to moderate cost	30 minutes	0.858 ± 0.006 ng/mL	[8]
**LC/MS/MS**	High cost	39 minutes	7.5 µg/L	[9]
**HPLC-UV**	High cost	17 minutes	902 ng/mL	[10]
**Colloidal gold immunochromatography assay**	Low to moderate cost	15 minutes	30-50 µg/kg	[11]
**Enzyme-linked immunosorbent assay (ELISA)**	Low to moderate cost	90 minutes	50 µg/kg	[11]
**Multiplex dipstick immunoassay**	Low to moderate cost	10 minutes	0.1 ng/mL	[12]
**UFLC-MS/MS and GC–MS/MS**	High cost	40 minutes	1 ng/g	[13]
**Colorimetric aptasensor**	Low to moderate cost	50 minutes	0.039 µg/mL	[14]
**Luminescence-based microbial method**	Low to moderate cost	-	6.3 ng/mL	[15]
**SERS platform**	Low cost	-	< 0.1 µg/mL	[16]
**SERS platform**	Low cost	10 minutes	0.07 µg/mL	This work

References

(1) Polavarapu, L.; Porta, A. L.; Novikov, S. M.; Coronado-Puchau, M.; Liz-Marzán, L. M. Pen-on-Paper Approach Toward the Design of Universal Surface Enhanced Raman Scattering Substrates. *Small* **2014**, *10* (15), 3065–3071. https://doi.org/10.1002/smll.201400438.

(2) Gill, R. A. Fillers for Papermaking. In *Applications of Wet-End Paper Chemistry*; Au, C. O., Thorn, I., Eds.; Springer Netherlands: Dordrecht, 1995; pp 54–75. https://doi.org/10.1007/978-94-017-0756-5_4.

(3) Sanz, J.; Tomasa, O.; Jimenez-Franco, A.; Sidki-Rius, N. Calcium Carbonate (Calcite). In *Elements and Mineral Resources*; Sanz, J., Tomasa, O., Jimenez-Franco, A., Sidki-Rius, N., Eds.; Springer Nature Switzerland: Cham, 2022; pp 341–343. https://doi.org/10.1007/978-3-030-85889-6_85.

(4) Fishkin, A. *UV-Vis Spectrum of Crystal Violet*. SIELC Technologies. https://sielc.com/uv-vis-spectrum-of-crystal-violet.

(5) Cañamares, M. V.; Chenal, C.; Birke, R. L.; Lombardi, J. R. DFT, SERS, and Single-Molecule SERS of Crystal Violet. *J. Phys. Chem. C* **2008**, *112* (51), 20295–20300. https://doi.org/10.1021/jp807807j.

(6) Harraz, F. A.; Ismail, A. A.; Bouzid, H.; Al-Sayari, S. A.; Al-Hajry, A.; Al-Assiri, M. S. Surface-Enhanced Raman Scattering (SERS)-Active Substrates from Silver Plated-Porous Silicon for Detection of Crystal Violet. *Applied Surface Science* **2015**, *331*, 241–247. https://doi.org/10.1016/j.apsusc.2015.01.042.

(7) Ali, S. K.; Salihu, S.; Olaleye, A. A. Development and Validation of Reverse - Phase HPLC Method for Detection of Tetracycline Residue in Fresh Milk Samples. *dujopas* **2023**, *9* (3a), 41–48. https://doi.org/10.4314/dujopas.v9i3a.5.

(8) Conzuelo, F.; Campuzano, S.; Gamella, M.; Pinacho, D. G.; Reviejo, A. J.; Marco, M. P.; Pingarrón, J. M. Integrated Disposable Electrochemical Immunosensors for the Simultaneous Determination of Sulfonamide and Tetracycline Antibiotics Residues in Milk. *Biosensors and Bioelectronics* **2013**, *50*, 100–105. https://doi.org/10.1016/j.bios.2013.06.019.

(9) De Ruyck, H.; De Ridder, H. Determination of Tetracycline Antibiotics in Cow's Milk by Liquid Chromatography/Tandem Mass Spectrometry. *Rapid Comm Mass Spectrometry* **2007**, *21* (9), 1511–1520. https://doi.org/10.1002/rcm.2991.

(10) Zergiebel, S.; Ueberschaar, N.; Seeling, A. Development and Optimization of an Ultra-Fast Microextraction Followed by HPLC-UV of Tetracycline Residues in Milk Products. *Food Chemistry* **2023**, *402*, 134270. https://doi.org/10.1016/j.foodchem.2022.134270.

(11) FODOR, A.; CSEPPENTO, D. C. N.; BADEA, G. E.; PETREHELE, A. I.; GROZE, A.; TIT, D. M.; BUNGAU, S. G. Colloidal Gold Immunochromatography and ELISA Traceability of

Tetracycline Residues from Raw Milk to Its Dairy Products. *In Vivo* **2023**, *37* (4), 1619–1627. https://doi.org/10.21873/invivo.13247.
(12) Han, S.; Zhou, T.; Yin, B.; He, P. A Sensitive and Semi-Quantitative Method for Determination of Multi-Drug Residues in Animal Body Fluids Using Multiplex Dipstick Immunoassay. *Analytica Chimica Acta* **2016**, *927*, 64–71. https://doi.org/10.1016/j.aca.2016.05.004.
(13) Jadhav, M. R.; Pudale, A.; Raut, P.; Utture, S.; Ahammed Shabeer, T. P.; Banerjee, K. A Unified Approach for High-Throughput Quantitative Analysis of the Residues of Multi-Class Veterinary Drugs and Pesticides in Bovine Milk Using LC-MS/MS and GC–MS/MS. *Food Chemistry* **2019**, *272*, 292–305. https://doi.org/10.1016/j.foodchem.2018.08.033.
(14) Luo, Y.; Xu, J.; Li, Y.; Gao, H.; Guo, J.; Shen, F.; Sun, C. A Novel Colorimetric Aptasensor Using Cysteamine-Stabilized Gold Nanoparticles as Probe for Rapid and Specific Detection of Tetracycline in Raw Milk. *Food Control* **2015**, *54*, 7–15. https://doi.org/10.1016/j.foodcont.2015.01.005.
(15) Kurittu, J.; Lönnberg, S.; Virta, M.; Karp, M. Qualitative Detection of Tetracycline Residues in Milk with a Luminescence-Based Microbial Method: The Effect of Milk Composition and Assay Performance in Relation to an Immunoassay and a Microbial Inhibition Assay. *Journal of Food Protection* **2000**, *63* (7), 953–957. https://doi.org/10.4315/0362-028X-63.7.953.
(16) Marques, A.; Veigas, B.; Araújo, A.; Pagará, B.; Baptista, P. V.; Águas, H.; Martins, R.; Fortunato, E. Paper-Based SERS Platform for One-Step Screening of Tetracycline in Milk. *Sci Rep* **2019**, *9* (1), 17922. https://doi.org/10.1038/s41598-019-54380-y.
